# There Is No Free Won’t: Antecedent Brain Activity Predicts Decisions to Inhibit

**DOI:** 10.1371/journal.pone.0053053

**Published:** 2013-02-13

**Authors:** Elisa Filevich, Simone Kühn, Patrick Haggard

**Affiliations:** 1 Institute of Cognitive Neuroscience, University College London, London, United Kingdom; 2 Department of Experimental Psychology and Ghent Institute for Functional and Metabolic Imaging, Ghent University, Gent, Belgium; 3 Centre for Lifespan Psychology, Max Planck Institute for Human Development, Berlin, Germany; University of Gent, Belgium

## Abstract

Inhibition of prepotent action is an important aspect of self-control, particularly in social contexts. Action inhibition and its neural bases have been extensively studied. However, the neural *precursors* of free decisions to inhibit have hardly been studied. We asked participants to freely choose to either make a rapid key press in response to a visual cue, or to transiently inhibit action, and briefly delay responding. The task required a behavioural response on each trial, so trials involving inhibition could be distinguished from those without inhibition as those showing slower reaction times. We used this criterion to classify free-choice trials as either rapid or inhibited/delayed. For 13 participants, we measured the mean amplitude of the ERP activity at electrode Cz in three subsequent 50 ms time windows prior to the onset of the signal that either instructed to respond or inhibit, or gave participants a free choice. In two of these 50 ms time windows (−150 to −100, and −100 to −50 ms relative to action onset), the amplitude of prestimulus ERP differed between trials where participants ”freely” chose whether to inhibit or to respond rapidly. Larger prestimulus ERP amplitudes were associated with trials in which participants decided to act rapidly as compared to trials in which they decided to delay their responses. Last-moment decisions to inhibit or delay may depend on unconscious preparatory neural activity.

## Introduction

Decisions for action can be decomposed into at least three separate functional components [1], associated with the selection of what action to make (*what* component); when to make it (*when* component) and *whether* to make it at all. The *whether* component is related to last-minute inhibition of an action that has been prepared and is ready for execution. This component may be particularly important as a mechanism for self-control [2]. These different forms of decision (*what, when, whether*) may be linked to different underlying neural processes.

Previous studies have linked preparatory activity preceding voluntary action to decisions about what action to make e.g., [3]–[5], or *when* to make it [6], [7]. Both these components of voluntary decision were shown to have unconscious neural precursors. First, decisions about when to act can bee associated with the readiness potential (RP), an accepted marker of neural preparation for action [6], [8]. Libet [9] famously identified RPs already occurring around 200 ms prior to the conscious decision to move (*when* component). Second, Soon et al, [10] found that brain activity several seconds before conscious decision could predict which hand people chose to act with (*what* component). However, the decision about *whether* to act has received less attention. Such *whether* decisions can be taken at almost any stage during motor preparation, up until a point of no return [11]. Libet controversially suggested that last-minute decisions to inhibit action involved a purely conscious form of “free won’t”. But theoretical grounds suggest that conscious decisions to inhibit must depend on unconscious brain processes, just like decisions to act [12]. However, neural precursors of voluntary inhibition have not yet been identified experimentally.

We report an experiment in which participants had to either make a rapid key press action, or *transiently* inhibit executing the key press, so as to briefly delay their response. In this way we operationalized inhibition as a transient process, characterised by delayed responding, rather than as a complete suppression of all behavioural output. Our operational definition has the advantage of matching the action and inhibition conditions more closely, since both conditions include a motor response – though with differing latencies. In everyday life, such impulse control by delaying a voluntary response may help in accumulating further information about the environment prior to responding [13], or in synchronising a joint action [14].

We compared neural activity preceding free decisions to act or briefly inhibit action. In free choice conditions, participants were not explicitly instructed whether to act rapidly or to delay in any given trial, but rather chose for themselves. In the absence of any external cause to act rapidly or inhibit, we hypothesized that some other factors, such as transient fluctuations in participants’ brain states, may be relevant to their decision in this case. Therefore, the free-choice condition would provide a situation in which putative internal fluctuations could lead to an overt modification of behaviour. We also reasoned that external instructions about action would produce a stronger drive of neural activity, overriding any intrinsic fluctuations. Consequently, we also compared neural activity preceding external instructions to act or to briefly inhibit action. Several recent studies suggest that the instantaneous state of the brain at the time when a new information-processing operation begins can play an important role in how information is processed. For example, the probability of remembering an item depends on preceding electrical neural activity [15], and the probability of detecting a visual stimulus depends on the phase of EEG alpha rhythm over frontocentral brain regions [16]. By analogy, we hypothesized that ‘free’ decisions to act or inhibit would depend on the progression of preceding activity in the brain.

The experiment followed a factorial design in which the *differences* in neural activity between free decisions to act and free decisions to inhibit were compared with the *differences* in neural activity between instructed decisions to act and instructed decisions to inhibit. We assumed that sorting trials according to action or inhibition might reveal patterns of preceding neural activity that might putatively cause the ‘free’ decision. In instructed decisions, in contrast, the cause of the decision to act or inhibit lied in the stimulus, rather than any putative pattern of preceding neural activity.

We measured electroencephalographic (EEG) activity around the time of an external instruction to either act quickly or delay transiently an action, or around the time of a cue that invited participants to choose to either act quickly or delay transiently. Although ERP methods do not typically provide high spatial resolution, they do provide high temporal resolution, [17]. This makes ERP methods particularly suited for our purposes, as they allowed us to identify neural activity preceding an instruction that influenced ”free” decisions in response to the instruction.

## Materials and Methods

Fourteen naïve healthy volunteers (9 females, mean 24 years, 12 right-handed) participated in this experiment. Before further data analysis, one participant was excluded from EEG analyses due to excessive eye blinking, leaving a total of 13 participants. Each participant did 8 blocks of 70 trials each, yielding a total of 560 trials.

Each trial fell into one of five possible experimental conditions. Trials could either be instructed rapid, instructed delayed, free-choice rapid, free-choice delay, or nogo trials.

**Figure 1:**
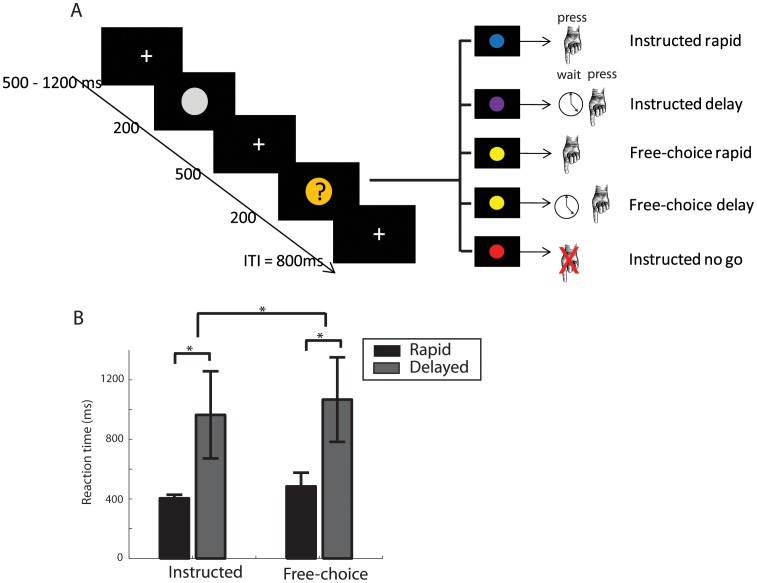
Experimental paradigm **A.** A variable fixation cross period (500–1200 ms) was followed by a brief (200 ms) presentation of a warning sign. The fixation cross reappeared and 500 ms after the offset of the warning sign, an instruction cue appeared on the screen (200 ms). The instruction cued participants to press a key either rapidly, or with a short delay, or to freely decide between rapid and delayed pressing. **B.** Instructed rapid and delayed trials are separated by their reaction times and free choices. Median split of free-choice trials produces a similar separation. Further, free choice trials are slower than instructed choice trials, suggesting a free decision process occurring after the cue. * p<0.05.

Each trial began with a variable fixation cross period (500 to 1200 ms, see fig 1A). A warning sign (a grey circle subtending 1.5°, duration 200 ms) appeared first. The fixation cross reappeared for 500 ms and was followed by an instruction cue (a coloured circle, 1.5° visual angle, 200 ms duration). The instruction cue indicated the trial type. In the instructed rapid condition (240 trials, 43%), participants were asked to press a key with their index finger as quickly as possible. In the instructed delayed condition (80 trials, 14%), participants had to make the same movement but with the “shortest possible delay”. The exact duration of the delay was not explicitly specified to the participants, but they were encouraged to delay their action for a period of time that was “as short as possible”. In the free-choice condition (160 trials, 28%), participants saw a cue that indicated that they were free to choose which action outcome to take. Namely, immediately upon the appearance of the free-choice cue, participants were asked to decide freely whether to act rapidly or after the shortest possible delay. In this way, the experiment followed a 2×2 factorial design, with the factors source of decision (instructed/free choice) and outcome (rapid/delayed). In addition, there was a further additional control condition, the nogo condition, in which participants were asked to refrain from acting (80 trials, 14%). The purpose of the nogo condition was to make the task more demanding and prevent drifts of attention. Data from nogo trials were not analyzed. The percentages of trials were constant across all blocks.

The rationale behind the experimental design was as follows. Neural networks continually exhibit small fluctuations in state, which may have significant effects on behaviour [18]. These effects may be particularly relevant for behaviour in the absence of other clear, strong external signals. We aimed to identify possible effects of such intrinsic fluctuations on the ‘free’ choice between action and transient inhibition. We assumed that similar intrinsic fluctuations should exist before instructed choices to act rapidly or delay actions. However, the strong signals linked to external instructions should override these weak internal signals, so that no differences between activity preceding actions and inhibition should remain in instructed conditions. Therefore, in a factorial design we compared neural activity prior to the decision between rapid or delayed action, where this decision was based either on internal free choice, or on external instructions. We expected to find differences in the neural activity preceding free-choice action decisions, because we hypothesized that preceding neural activity would strongly influence the decision between different possible action outcomes. We expected to find no differences in preceding activity between rapid and delayed action following external instructions, since the instructional signal should then have a far stronger influence on behavioural outcome.

In experiments involving freely-chosen inhibition, there is a high risk of participants deciding in advance not to make an action [19]. In cases of early decisions not to act, no action will be prepared, and consequently no action inhibition will be necessary. Therefore, tasks addressing freely-chosen inhibition should encourage action preparation. In this case, we included a high number of rapid instructed trials to encourage action preparation, to make delaying effortful, and to discourage participants from deciding in advance whether to respond rapidly or inhibit and delay on free-choice trials. Further, only for trials in the instructed rapid condition, participants were rewarded (3p) for every key press that was faster than their average in the previous block. The experimental design was therefore not strictly balanced, but emphasized the need for true action inhibition.

In free choice trials, participants were asked to balance their choices between rapid and delayed responses. The hand used for responding was fixed for each block, and alternated between blocks. The correspondence of colours to instructions rotated across participants, and was additionally reversed for each participant for the second half of the experiment. Trials within each participant were randomized, but the proportion of trial types was valid for each block.

Trials with RTs below 200 ms were rejected, as potentially anticipatory (1.76±1.67%). The average commission error rate in nogo trials was 10±0.9%. These trials were included merely to engage attention, and to ensure that participants responded only after receiving the Go signal. Nogo trials were not further analyzed. RTs for each condition were analyzed in a 2×2 repeated measures ANOVA, with the factors decision source (instructed/free-choice) and response speed (rapid/delay).

To examine whether participants followed any obvious strategy to produce a balanced outcome between rapid and delayed free-choice trials, we evaluated run length in the response sequences in free-choice trials. Our experiment consisted of 8 blocks, with 20 free-choice trials each. For each block, we excluded the instructed trials and measured the length of runs (i.e., sequences of uninterrupted repetitions of the same outcome) for each participant. If participants had been producing obvious sequences such as ‘AABBAABB’ they would produce a single run-length only (in this case, a run-length of 2). We then generated true random data. We generated 8 independent “blocks” of 20 “trials” each by sampling without replacement from a population of 10 quick trials and 10 delay trials. This restriction of a balance between quick and delay trials was necessary because our free-choice data was based on a median split, yielding balanced numbers of trials in the experimental data.

### Ethics Statement

Procedures were approved by the UCL research ethics committee and were in accordance with the principles of the Declaration of Helsinki and written informed consent was obtained from all participants involved in the study.

### EEG Data Acquisition and Analysis

A SynAmps amplifier system and Scan 4.3 software (Neuroscan, El Paso, TX) were used to record EEG data. We recorded activity from fourteen scalp electrodes (F3, Fz, F4, FC3, FCz, FC4, C3, Cz, C4, P3, Pz, P4, O1, O2) and the right and left mastoids. The scalp electrodes were placed according to the international 10–20 system. The reference electrode was AFz and the ground electrode was placed on the chin. All electrode impedances were kept below 5 KΩ. Electroculograms (EOG) were recorded from bipolar electrodes placed on the left and right external canthi (to detect horizontal eye movements), and on the right supra-orbital and infra-orbital positions (to detect vertical eye movements). EEG signals were amplified and digitized at 500 Hz.

EEG data were analyzed with EEGLAB software (Delorme and Makeig, 2004). Data were first re-referenced to the linked mastoids. Data were digitally band-pass filtered between 0.05 Hz and 30 Hz. Continuous EEG data was time-locked to the instruction stimulus, and epochs were defined from −850 ms to 700 ms after the instruction sign. A baseline period was defined for each epoch from −850 to −700 ms (between 0 and 150 ms prior to the onset of the warning signal). The hand required for action was alternated and specified at the beginning of each block. Lateral (non-midline) electrodes were inverted in the right hand blocks, as if all data had been collected from the *left* hand. Because we inverted the lateral electrodes from the right hand blocks, electrodes represented in the left hemisphere are now ipsilateral to action. Similarly, electrodes represented in the right hemisphere are contralateral to action.

Right-left hand symmetry cannot be assumed in this situation. First, the left hemisphere is dominant for action preparation [20]. Second, whereas RPs associated with right hand movements are normally distributed, this is not the case for left hand movements [21]. The distribution of the early left-hand movement RP amplitudes shows negative skewness values, even in cases of very simple actions, such as key presses with the index finger. This suggests that movements with the non dominant hand may require more attentional resources and/or special preparatory processes.

To remove blink artifacts, epochs were rejected if the difference between the two vertical EOG channels was larger than 90 µV.

For ERP data analysis, three consecutive 50 ms time windows prior to the instruction cue were defined (−150 to −100 ms, −100 to −50 ms and −50 to 0 ms). These timepoints were selected based on previous studies on prestimulus ERP activity [15]. The mean EEG amplitude in the electrode Cz was calculated for each participant. As in the case of the RTs, mean window ERP amplitudes were analyzed in repeated measures ANOVA, using the statistical packages SPSS for IBM-PC (SPSS Inc.) and custom Matlab functions (The Matworks, Inc.). Greenhouse-Geiser (GG) corrections were applied when appropriate, but full degrees of freedom are reported.

Our experimental design included a much larger number of trials in the instructed rapid condition than in the other three conditions. We therefore used resampling methods to control for uneven numbers of trials [22]. For each participant, the number of trials in the instructed delay condition was found. The same number of trials was then randomly sampled, with replacement, from the trials in the instructed rapid condition. These two populations of trials were then combined to get an overall distribution of instructed RTs. A trial was considered as correct in the instructed rapid condition if its RT was quicker than the median of the distribution of instructed RTs. In the same way, a trial was considered as correct in the instructed delayed condition if its RT was slower than the median of the distribution of instructed RTs. Finally, the mean CNV amplitude measured from electrode Cz was obtained for all four main trial types, in each of the 50 ms time windows prior to the instruction, and averaged across subjects. This procedure was repeated 10,000 times.

## Results

### Behavioural Results

Following the monetary reward incentive to the instructed rapid trials, participants became quicker in each block. The total number of rewarded trials (i.e., those instructed rapid trials that were quicker than the average of the previous block) was 156±10 (mean ± SD), and there was a mean decrease in RT of 55 ms.

Instructed trials were classified as rapid or delayed *a priori*, according to the instruction given in each trial. Free-choice trials lacked a specific instruction, and hence were classified as rapid or delayed *a posteriori*, on the basis of a median split of each participant’s free-choice response RT distribution (figure 1B, see below for sensitivity analysis). ‘Because the free choice trials were classified as rapid or delayed (i.e., median split), exactly half of the trials were rapid, and half of the trials were delayed.

To determine the effect of the decision in the free-choice conditions, we performed a 2×2 ANOVA on the RTs with the factors decision source (instructed/free-choice) and response outcome (rapid/delay). The main effect of source of decision (F_1,13_ = 7.15; p = 0.019) arose because free-choice responses were slower than instructed responses. This suggests that the free decision to respond rapidly or to transiently inhibit and delay involved a time-consuming cognitive process occurring after the instruction signal. The main effect of outcome (F_1,13_ = 81.43; p<0.001) unsurprisingly showed that participants significantly delayed their RTs both in instructed and in free-choice conditions. There was no significant interaction between source of decision and outcome (F_1,13_ = 0.12; p = 0.734).

Control behavioural analyses were performed on the data from the 13 participants considered in the EEG measurements. Participants switched hands in each block. Therefore there could have been an effect of hand used. To examine this possibility we compared the RTs for blocks in which participants used their dominant *vs.* nondominant hand. We conducted a repeated measures three-way ANOVA, with the factors block subset (dominant hand/nondominant hand blocks), source (instructed/free-choice) and outcome (rapid/delayed). We found a main effect of block subset (F_1,12_ = 6.40, p = 0.026), indicating that participants were quicker to make actions with their dominant hands, as might be expected. However, there was no significant three-way interaction (F_1,12_ = 0.08 p = 0.770), indicating that the hand used did not affect the interaction of source x outcome that is of interest here.

Similarly, to rule out low-level effect of the physical stimuli, the correspondence between S2 colour and instruction was changed half way through the experiment. This could have led to a significant Stoop-like effect [23]. To explore this possibility, we again conducted a repeated measures three-way ANOVA, with the factors block subset (first half/second half), source (instructed/free-choice) and outcome (rapid/delayed). We found a main effect of block subset (F_1,12_ = 18.38, p = 0.01), suggesting that participants had learnt the association in the first half of the experiment, and the switch in association between colour of the instruction cue and the task generated a Stroop-like effect. Importantly however, as in the case of hand used we found no significant three-way interaction (F_1,12_ = 0.47, p = 0.505), suggesting that the source x outcome interaction was not modulated by Stroop-like effects.

### Participants’ Strategies

We asked participants to produce roughly 50% rapid and 50% delay responses. This may have led to stereotyped behaviour, such as chunking or direct alternating strategies. If this had been the case, the decision to act rapidly or delay would not have been taken just before the instruction, but presumably at the onset of the trial. To discourage this strategy, we interleaved instructed and free-choice trials. The alternation between free rapid and delayed trials would therefore require a higher effort of maintenance of the preceding history of choices in working memory. We did formal tests to rule out potential chunking behaviour (e.g., patterns of responses such as AABBAABB). We examined the run length in each participants’ sequence of free-choice responses, and compared it with simulated random data (see figure S1 in Supporting information S1). The simulated data shows the same pattern than the experimental data. To test if this was indeed the case, we did a 2×4 ANOVA with the factors data type (experimental/simulation) and run length (1 to 4). We found a main effect of data type (F_1,13_ = 6.98, p = 0.02) and a significant data type x run length interaction (F_2,26_ = 4.19, p = 0.019). We therefore did independent paired t-tests for the number of runs of length 1,2,3 and 4. We found that only the number of run lengths of 1 differed significantly between the experimental and the simulated data (experimental data mean ± SD: 40±6 runs; simulated data: 46±6 runs; t_13_ = −2.981, p = 0.010). Participants showed fewer runs of length 1 than expected based on simulation results, indicating that subjects tended to avoid direct alternation.

Finally, to statistically test for randomness of responses we collapsed all blocks of each subject into a single run of 160 trials, we performed a Wald-Wolfowitz Runs test [24] for each subject. The null hypothesis that the sequence generated was random was not rejected for any participant (all p>0.135).

### ERP Results

After artefact rejection, an average of 164±70 trials (69% of original trials) remained for the instructed rapid condition, 67±12 (84%) trials for the instructed delayed condition, 57±22 (72%) trials for the free-choice rapid condition and 69±9 (86%) trials for the free-choice delayed condition. Participants were told that they could blink only after having made an action, to prevent the well-known tendency to blink and press the key at the same time. This instruction could explain the observed differences in the proportion of rejected trials between action and transient inhibition conditions Earlier key presses in the rapid conditions might have been the cause of more blinks occurring during the epoch of interest.

**Figure 2:**
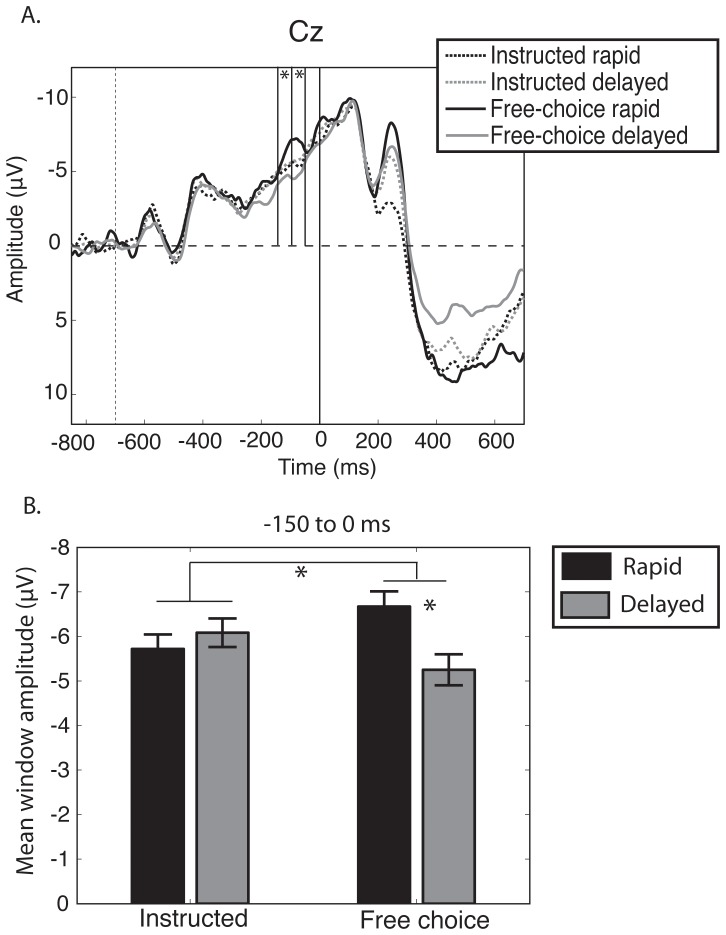
ERPs time locked to the instruction cue **A.** Averaged CNV amplitude in electrode Cz for the four main conditions, time locked to the appearance of the instruction cue (time 0 corresponds to the onset of the instruction cue). Note the difference in CNV amplitude between two free-choice rapid and delayed trials (solid lines), but no difference in CNV amplitude between instructed choice rapid and delayed trials (dashed lines). Asterisks indicate a significant ANOVA interaction (*F* test, p<0.05, uncorrected). Vertical dashed line at −700 ms indicates onset of warning signal and the end of baseline period (−850 to −700 ms). **B.** mean CNV amplitudes for the time window between −150 and 0 ms electrode Cz. Asterisk shows a significant difference (t-test, p<0.05, two tails, uncorrected).

Event-related potentials (ERPs) showed a clear negativity before the instruction signal (Figure 2). This corresponds to the classical contingent negative variation (CNV), [25], [26].

**Figure 3:**
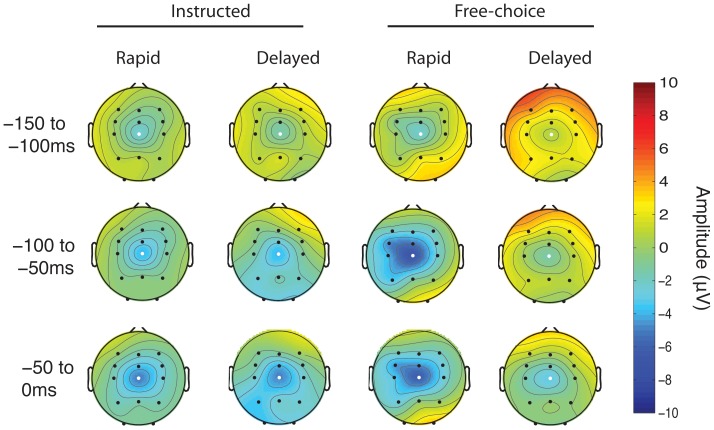
Topographical plot of ERP amplitude before the instruction cue. Topographical distribution of the CNV component for each of the four main conditions, averaged over three time windows selected for analysis. White highlight shows electrode Cz, from which the mean time window amplitudes were obtained for statistical analyses.

To examine the topography of this component, we produced scalp maps in the three time windows of interest. These maps show that the CNV shows a broad distribution, centred on electrode Cz (see figure 3).

To explore differences in the CNV amplitude between conditions, we first explored the topography of the CNV potential. We conducted a 2×2×3 ANOVA, with the factors source (instructed/free choice), outcome (rapid/delay) and electrode group (ipsilateral/midline/contralateral). Segmenting electrodes into regions rather than entering them individually as factors into an ANOVA is a more informative approach [17]. We excluded the parietal and occipital electrodes, given the a priori hypothesis of the known topographical distribution of the CNV [25], [26]. To simplify the analyses, we chose the single time window of −150 to 0 ms prior to the instruction cue.

We found a main effect of electrode group (F_2,24_ = 8.59, p = 0.002), no main effect of source (F_1,12 = _0.03, p = 0.874) and no main effect of outcome (F_1,12_ = 0.95, p = 0.348). We found a marginally significant source x outcome interaction effect (F_1,12_ = 4.55, p = 0.054). This effect did not interact with electrode group (F_2,24_ = 0.24, p = 0.673). We therefore took the standard approach of using the electrode Cz for the analysis of the CNV amplitude.

**Figure 4:**
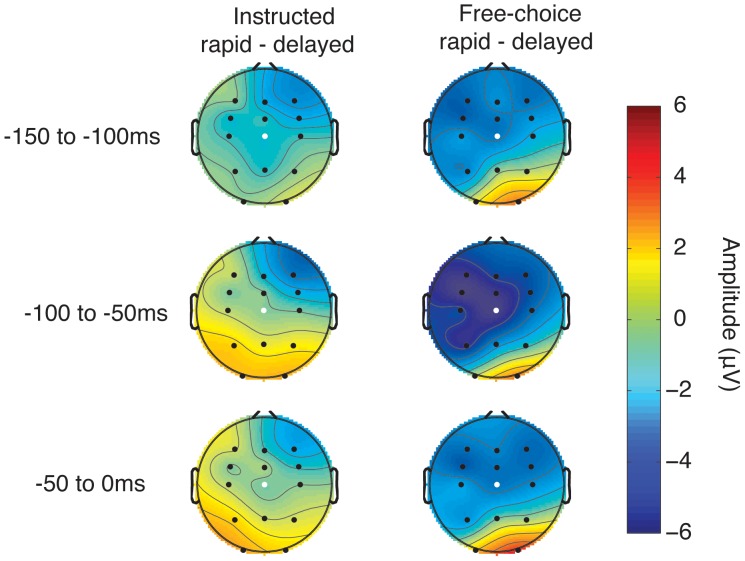
Differential topographical plots of ERPs. Topographical plots of the difference in brain activity between rapid and delayed trials. Depicted values are averaged amplitudes over 50 ms time windows. Note stronger difference in free-choice than in instructed conditions. White highlight shows electrode Cz, from which the mean time window amplitudes were obtained for statistical analyses.

We next explored whether there were any differences between conditions over the three time windows defined for analysis. We used a 2×2×3 ANOVA with the factors source, outcome and time bin (−150 to −100 ms/−100 to −50 ms/−50 to 0 ms). We found a main effect of time window (F_2,24_ = 8.77, p = 0.007); no main effect of source (F_1,12_ = 0.03, p = 0.862) and no main effect of outcome (F_1,12_ = 1.16, p = 0.302). There was a significant interaction effect between source and outcome (F_1,12_ = 6.06, p = 0.030). This interaction was explored by post-hoc testing. In the free choice condition, the CNV amplitude measured from Cz was reduced (i.e., less negative) when participants chose to transiently inhibit and delay than when they chose to respond rapidly. In contrast, the instructed condition showed no difference between rapid and delay trials. That is, CNV amplitude just before the decision cue had a specific association with subsequent free choices to respond rapidly or to delay. Figure 4 shows the topographical distribution of these differences.

This two-way interaction in turn shows a marginally significant interaction with time window, as shown by the three-way interaction in ANOVA, F_2,24_ = 3.8, p = 0.051). Because of this marginal three-way interaction, we evaluated the source x outcome interaction effect in each one of the three time windows (−150 to −100 ms, −100 to −50 ms and −50 to 0 ms). We found the source x outcome interaction in the −150 to −100 ms (p = 0.041) and −100 to −50 ms window (p = 0.016), but not the −50 to 0 ms window (p = 0.110) (see table 1). Because post-hoc t-tests were examined only to explore significant interactions, corrections for multiple comparisons were not used.

**Table 1:** Results of statistical analyses of ERP amplitudes in three 50 ms time bins.

	Interaction		Free-choice		Instructed	
Time interval (ms)	F_1,12_	p	t_12_	p	t_12_	p
**−150 to −50**	5.18	0.041	−1.79	0.097	0.58	0.56
**−100 to −50**	7.75	0.016	−2.57	0.024	0.66	0.515
**−50 to 0**	2.96	0.110	−0.80	0.437	1.05	0.313

The table shows the interaction term of a 2×2 ANOVA (source of decision x outcome), and results of the follow-up t-tests. All p values are uncorrected. See text for details.

We classified rapid and delayed trials *a priori* in the instructed condition, but *a posteriori* in the inhibit/delayed condition. We thus assumed that *instructed* rapid and *instructed* delayed trials were drawn from separate populations, with different mean RTs. However, if participants had completely failed to follow the instruction to respond rapidly, or with a delay, then instructed rapid and instructed delay RTs would not have differed. In the CNV, RT and ERP amplitude have been shown to be inversely related [27]. The interaction we found between instructed and free-choice conditions could then be an artifact of using *a priori* classification criteria for instructed conditions, but *a posteriori* classification criteria for free-choice conditions.

We suggest this is not the case, for several reasons. First, a strong main effect of outcome emerged when instructed trials were classified *a priori* according to the instruction signal, suggesting that participants indeed attended to the instruction to respond rapidly or to delay, and indeed generated two distinct populations of instructed trials with minimal overlap in RT. Crucially, we found no significant interaction (p = 0.73) between decision outcome and decision source. This was also the case when we controlled for hand used or possible confusion due to Stroop-like effects. These findings suggest that participants were equally able to produce distinct rapid and delayed actions in instructed and free-choice conditions. Thus, our *a priori* criterion for instructed trials and *a posteriori* criterion for free-choice trials were approximately matched. Since treatment of RTs was successfully matched across instructed and free-choice conditions, differences between ERP amplitudes cannot simply be a consequence of differences in RT distributions.

**Figure 5:**
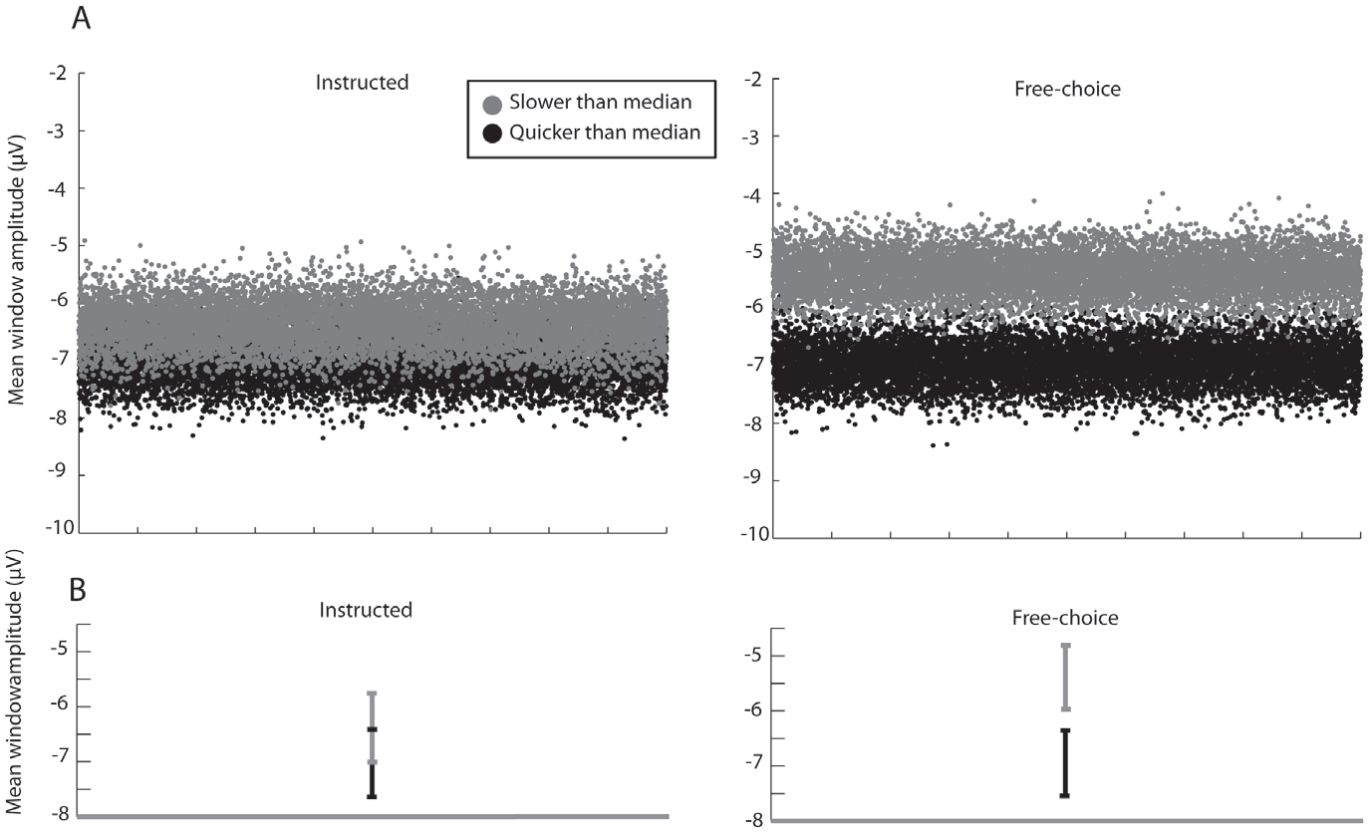
Control to account for differential trial numbers between conditions. Bootstrapping procedure to resample instructed trials, compensating for differences in numbers of each trial type. This procedure allows instructed and free-choice reactions to be classified based on reaction times. Results are shown for the interval of −150 to 0 ms prior to the onset of the visual cue, in electrode Cz **A.** The instructed rapid and delayed subsampled populations cannot be easily distinguished. In contrast, the free-choice rapid and delayed subsample populations are clearly distinct. **B.** 95% Confidence intervals for instructed and free-choice conditions. Note that while they are separate in the free choice conditions, they overlap in the instructed conditions.

Second, we performed an additional analysis in which instructed trials and delayed trials were *both* classified in the same way, using an *a posteriori* criterion, based on RT. Our experimental design deliberately over-emphasised the number of instructed rapid trials. To account for possible overestimation of differences due to an *a posteriori* criterion, we used subsampling methods (See Methods section). Results of this subsampling procedure are shown in figure 5.

If participants had ignored the instruction signal, the mean proportion of correct instructed trials should have been around 50% in both conditions. Instead, the mean proportion of correct trials was 87.1±9% in the rapid condition; and 87.2±8% in the delay condition. This suggests that the *a priori* classification yielded similar populations than the *a posteriori* classification.

In the case of free-choice conditions, the resampled data formed two clearly distinct populations. One population of resampled trials presented slower, above-median RTs and was therefore classified as delayed/inhibited. These trials were associated with lower prestimulus CNV amplitudes. A second population presented faster, below-median RTs. These trials were thus classified as rapid responses and were associated with higher prestimulus CNV amplitudes. The 95% confidence intervals for the two resampled populations did not overlap, replicating the finding of our main analysis. Prestimulus CNV amplitude differed before a free choice to respond rapidly or with a delay. In the instructed conditions, the resampled data did not form two clearly distinct populations and the 95% confidence intervals for prestimulus CNV amplitude show clear overlap between slower, above-median RTs classified as delayed/inhibited, and faster below-median RTs classified as rapid. The same resampling procedure was repeated for all three time windows (see supplementary material, figure S2 and table S1 in Supporting information S1).

Lastly, we analyzed the RT distribution for each participant (see supplemental figures S3 to S16 in Supporting information S2 file for individual distributions). Could the strong difference in the CNV amplitudes between the free-choice conditions be a simple consequence of a strong separation between quick and delayed RTs? If this were the case, then RT distributions in the free conditions should be more bimodal than instructed conditions. We used an established coefficient of bimodality *b* appropriate for large trial numbers [28]:
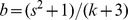
Where *s* and *k* are indexes of skewness and kurtosis respectively. The index of bimodality was in fact lower in the free-choice than in the instructed condition (the instructed conditions showed a higher coefficient of bimodality than free-choice conditions, mean ± SD 0.44±0.07 and 0.22±0.05, respectively). These measures of bimodality were significantly different (t_13_ = 9.7, p<0.01). The difference remained significant when data was subsampled using the same procedures as the one described for the CNV amplitudes. This provides further evidence against the possibility that CNV amplitude differences in the free-choice conditions simply reflect stronger RT differences for free than instructed choices.

## Discussion

In this experiment participants were instructed either to press rapidly or to inhibit and delay a key press; or they were free to choose between these two alternatives. Our results show that the neural activity before the moment of decision to inhibit differed from that before a decision to act rapidly. When participants chose to respond rapidly on free-choice trials, they did so on the basis of stronger preparatory activity *before* the moment of choice. Choosing to transiently inhibit and delay responding was associated with lower preparatory activity. This prestimulus influence on decision was unique to free-choice trials, and was absent or reduced when participants were instructed to inhibit/delay. By definition, in the instructed condition, participants’ behaviour was dictated by the instruction cue. Therefore the prestimulus CNV activity cannot predict instructed behaviour. Consequently we used the instructed condition as a negative control, and sought to find differences in the prestimulus CNV trace between the two free-choice conditions.

Because different criteria were used to classify rapid and delayed trials for instructed and free-choice trials, we performed additional analyses in which instructed trials were also classified according to their RTs. The pattern of results remained the same. Therefore, a specific prestimulus CNV amplitude difference between rapid and delayed actions was still present for free-choice trials, but not for instructed-choice trials, even when the number of trials was balanced across conditions, and classification criteria were chosen to distinguish rapid and delayed responses in a similar way for free-choice and instructed decisions.

Could our result have occurred because of variations in general arousal level? Specifically, a participant who was mindwandering or not engaged in the task might be expected to show low CNV amplitudes and long RTs [25]. Conversely, a high preceding level of arousal and engagement would be likely to produce a short RT. Thus, on a free-choice trial, a prior state of high arousal would be likely classified as a decision to respond rapidly, even if no specific cognitive process of decision actually occurred. Similarly a low preceding level of arousal would be likely to be classified as a decision to transiently inhibit responding. On this view, the relations between prior CNV activity and RT that we identified as decisions to act or inhibit might in fact be due to general arousal effects, rather than effects of prior neural activity on a specific cognitive decision process. However, the variation in RT in our data was much larger than that expected due to arousal effects alone. For example, Cheyne [29] have described the “natural” fluctuations in RT in a go/nogo task [30]. Their results show, for example, that trials preceding commission errors were on average 20 ms quicker than other trials. Conversely, trials preceding omission errors were on average 150 ms slower than the baseline. In our experiment, the differences between rapid and delayed trials were of around 600 ms, much longer than delays explained by occasional inattention or “zoning out” episodes. We argue that our RT differences reflected outcomes of a specific decision process, and that this specific process was driven by neural precursor activity. This precursor activity may well have been in turn related to arousal, but our effects were clearly mediated by a specific action/inhibition decision process. This decision process occurred either based on external instruction, or on participants’ “free” decisions. We show that these free decisions to inhibit/delay in fact depended on preceding brain activity, before the instruction to decide. The current state of the brain appears to influence the conscious decision to act or inhibit/delay, rather than vice versa.

Could our participants actually have decided to inhibit/delay *before* the visual signal to choose? Two facts argue strongly against this potential predecision. First, we included frequent and rapid-response trials in the instructed condition to discourage such early predecision, and rewarded participants according to their RTs on these trials. Second, a 2×2 ANOVA revealed a main effect of source of decision, with free-choice trials being 90 ms slower than instructed trials (p = 0.019), consistent with a time-consuming decision stage occurring after the instruction, and comparable to RT costs of instructed choices (Hick, 1952).

Finally, to discourage stereotyped behaviour in the free-choice trials (such as direct alternation between action outcomes), we interleaved instructed and free-choice trials. In this way, a predecided strategy to maintain a stereotyped behaviour would have required higher working memory load. To check whether such predecided strategies were followed, we examined the distribution of the length of runs (i.e., sequences of repeated action outcomes) for each participant. A distribution of runs strongly centred around a given number would have indicated a predecided strategy, No single participant showed evidence for a stereotyped behaviour.

These data suggest that free choices to inhibit/delay were made *after* the visual cue, but were strongly driven by antecedent, unconscious brain activity.

### Limitations of This Study

Several limitations should be considered when interpreting the results of this study. Our sample size is relatively low, and our inferences should therefore be taken with caution. Nevertheless, the size of our study is comparable with other recent studies on prestimulus EEG activity [31]–[33].

In terms of design and data analysis, five important limitations should be taken into consideration. First, our factorial design was not perfectly balanced, as it included a relatively higher number of instructed quick trials as compared to instructed delayed trials. Participants were rewarded on the basis of these rapid instructed trials only. At the end of each block they received a reward proportional to the number of rapid instructed trials that were faster than the average on the preceding block. Because only instructed rapid trials were rewarded, the free and instructed conditions could have differed in terms of motivation. These differences in motivation may have influenced the way in which movements have been prepared or delayed. This imbalance in both trial numbers and reward was the result of a strategic decision to try to discourage participants from predeciding before each trial whether to act or delay. By using the rewards on instructed trials to motivate advance preparation of actions, we could assume with greater confidence that delayed responses involved an inhibition of an already-prepared action.

Second, we classified free-choice trials as rapid or delayed actions based on their reaction times. This approach has the advantage of not relying on subjective report, but only on objective behavioural measures. However, these objective measures may not provide a perfect classification. Long RTs may be indicative of action inhibition, but may also arise for other reasons than inhibition, such as failures of attention, long decision times, etc. However, if our classification approach were simply imperfect, this would count against the probability of finding significant differences between trial types.

Arousal is one particular factor that might influence RT by affecting preparation. However, a *general* relation between arousal and RT would be presumably common to both instructed and free-choice conditions. To explore the particular possibility of a role of arousal, we conducted the resampling analysis splitting the instructed data into rapid and delayed based on the median RT. In this way, had arousal been the *only* factor influencing the CNV amplitudes, then the instructed conditions would have shown two different populations in our resampling analysis. This was not the case. Instead, we found a *specific* relation between preparatory activity and a free decision to delay, with no such relation in instructed choice conditions. This can not be explained by a general relation between arousal and RT without additional *ad hoc* assumptions.

A third limitation of our study comes from the low spatial resolution of ERP [17]. In particular, the differences in CNV amplitude that we found prior to rapid vs. delayed free-choice responses may have a subcortical source [34] that cannot be measured at the scalp.

Fourth, our analysis may miss out some hemisphere-specific variations in preceding neural activity. We asked participants to switch hands in every block, and then collapsed the ERPs obtained for the hemisphere contralateral and ipsilateral to the movement, regardless of the hand actually used for movement. However, the distribution of RPs in left and right hemisphere is known to differ (e.g., [35]). Dirnberger et al [21] have shown that there are “atypical” trials in left hand key press tasks (but not in right hand key press tasks). These trials were found to have exceptionally early pre-movement activity. Such atypical trials lead to RP amplitude distributions that violate the assumption of a Gaussian distribution, necessary for the parametric analyses used here. Because we collapsed trials made with the right and left hand, it is not clear whether such atypical trials are present here, and how valid the assumptions of normality are. However, our design focussed on differences between free-choice and instructed conditions, with equal numbers of right and left hand movements in each condition. To our knowledge, any bias introduced by hemispheric asymmetry should be equivalent in free-choice and instructed conditions, and would therefore not influence our conclusions. Nevertheless, further control experiments could check for potential right-left asymmetries.

Finally, as is common practice in paradigms involving free choices, we asked participants to try to balance their choices, and roughly choose to act rapidly in 50% of the free-choice trials. This requisite for a roughly balanced behaviour may have encouraged participants to predecide in advance the sequence of free action outcomes they would choose. We formally tested this possibility and found no evidence for non-random behaviour, but the possibility cannot be fully discarded.

### Implications of This Study

Free decisions about *what* action to make have been shown to be affected by subliminal primes [36]. In the same way, subliminal primes have been shown to modulate ERP components typically associated with inhibition in a go/nogo task [37]. Here we did not present subliminal primes to alter the preceding neural activity, but instead capitalized on the intrinsic variation in brain activity preceding the Instruction to decide. We argue that participants “freely decided” to respond quickly or delay their responses, depending on the degree of preparation within the cortical motor system immediately preceding the instruction to decide. Our data can be parsimoniously explained by the suggestion that ”conscious free decisions” to inhibit action may depend on the preceding state of the brain. Interestingly, the classic definition of voluntary actions involves contrasting them with instructed, stimulus-driven actions [38]. Volition thus amounts to “not externally generated” action. Our cortical excitability measures would presumably satisfy this definition, since they correspond to fluctuations of internal signals. Links between free will and other internal neural signals have been proposed, notably the default mode network [39].

Antecedent brain activity was shown to precede subsequent conscious decisions about when to act (by about 700 ms -Libet et al, 1983-), or to be predictive of what action to make (by several seconds -Soon et al, 2008-). EEG activity was also reported to precede “free decisions” to inhibit [40]. However, these results depend on interpreting *subjective* reports about time of free decisions, which remain controversial [41]. Moreover, the experimental designs of those studies did not take the steps we have taken to exclude advance pre-decision about whether to action or not. Our task was designed to constrain the decision to act or delay/inhibit to an identifiable point in time. This makes the finding of antecedent neural prediction of “free” decisions more striking, and may provide more convincing evidence for a form of neural determinism. In particular, our results show that antecedent brain activity influences “free choices”. This is true even when the decision process is precisely defined in time, and when data analysis is based on objective behavioural criteria, rather than on subjective reports.

Importantly, our results also illustrate that unconscious brain activity significantly influences behaviour in situations where participants internally generate for themselves how to respond, yet there is no strong motivation to choose any one possible response alternative over the other. Preceding brain activity may have much less influence on behaviour when a clear instruction or strong internal motivation (such as a financial incentive) encourages choosing one response alternative over the other. In that case, any influence of preceding brain activity will be diluted or overridden to produce the “correct” response. On the other hand, cases of decision without clear instruction or strong internal motivation are particularly important, because they are the focus of debates about “endogenous” decisions, and more generally about “free will”.

Our main argument is as follows: Libet et al, (1983) had suggested that decisions to inhibit action have an important role in freedom of will, because, he argued, they do not have any obvious unconscious neural precursors. In Libet’s view, this makes decisions to inhibit crucially different from decisions to act, for which, he claimed, there *is* a clear unconscious precursor. Libet’s dualistic notion of “free won’t” has been criticised on theoretical grounds. However, in our view, a stronger rejection of “free won’t” could come from actually showing that a decision to act or not can be driven by a preceding, presumably unconscious neural activity. Our results identify, for the first time, a candidate unconscious precursor of the decision to inhibit action. These results count as evidence against Libet’s view that the decision to inhibit action may involve a form of uncaused conscious causation.

### Conclusion

Neuroscience cannot straightforwardly accommodate a concept of “conscious free will”, independent of brain activity [42]. However, the belief that humans have free will is fundamental to human society [43]. This belief has profound top-down effects on cognition [44] and even on brain activity itself [45]. The dualistic view that decisions to inhibit reflect a special “conscious veto” or “free won’t” mechanism [46] is scientifically unwarranted. Instead, conscious decisions to check and delay our actions may themselves be consequences of specific brain mechanisms linked to action preparation and action monitoring [19]. Recent neuroscientific studies have strongly questioned the concept of free will, but have had difficulty addressing the alternative concept of free won’t, largely because of the absence of behavioural markers of inhibition. Our results suggest that an important aspect of “free” decisions to inhibit can be explained without recourse to an endogenous, ”uncaused” process: the cause of our “free decisions” may at least in part, be simply the background stochastic fluctuations of cortical excitability. Our results suggest that free won’t may be no more free than free will.

## Supporting Information

Supporting Information S1 **This file contains supporting figures, S1 and S2, and table S1.**
(DOCX)

Supporting Information S2 **This file contains supporting figures, S3 to S16, representing individual distributions of RTs.**
(DOCX)

## References

[1] Brass M, Haggard P (2008) The what, when, whether model of intentional action. Neuroscientist. 14, 319–325. 10.1177/1073858408317417 18660462

[2] Filevich E, Kühn S, Haggard P (2012) Intentional inhibition in human action: The power of “no.”Neurosci Biobehav Rev. 36, 1107–1118. 10.1016/j.neubiorev.2012.01.006 22305996

[3] Deiber MP, Passingham RE, Colebatch JG, Friston KJ, Nixon PD, et al.. (1991) Cortical areas and the selection of movement: a study with positron emission tomography. Experimental Brain Research. 84, 393–402. 10.1007/BF00231461 2065746

[4] Frith CD, Friston KJ, Liddle PF, Frackowiak RSJ (1991) Willed action and the prefrontal cortex in man: a study with PET. Proc. Biol. Sci. 244, 241–246. 10.1098/rspb.1991.0077 1679944

[5] Jueptner M, Frith CD, Brooks DJ, Frackowiak RSJ, Passingham RE (1997) Anatomy of Motor Learning. II. Subcortical Structures and Learning by Trial and Error. J Neurophysiol. 77, 1325–1337. 10.1152/jn.1997.77.3.1325 9084600

[6] Jahanshahi M, Jenkins IH, Brown RG, Marsden CD, Passingham RE, et al.. (1995) Self-initiated versus externally triggered movements: I. An investigation using measurement of regional cerebral blood flow with PET and movement-related potentials in normal and Parkinson’s disease subjects. Brain. 118, 913–933. 10.1093/brain/118.4.913 7655888

[7] Libet B, Wright EW, Gleason CA (1982) Readiness-potentials preceding unrestricted “spontaneous” vs. pre-planned voluntary acts. Electroencephalography and Clinical Neurophysiology. 54, 322–335. 10.1016/0013-4694(82)90181-x 6179759

[8] Dirnberger G, Fickel U, Lindinger G, Lang W, Jahanshahi M (1998) The mode of movement selection Movement-related cortical potentials prior to freely selected and repetitive movements. Experimental Brain Research. 120, 263–272. 10.1007/s002210050400 9629968

[9] Libet B, Gleason CA, Wright EW, Pearl DK (1983) Time of conscious intention to act in relation to onset of cerebral activity (readiness-potential). The unconscious initiation of a freely voluntary act. Brain. 106 (Pt 3), 623–642. 10.1093/brain/106.3.623 6640273

[10] Soon CS, Brass M, Heinze HJ, Haynes JD (2008) Unconscious determinants of free decisions in the human brain. Nature neuroscience. 11, 543–545. 10.1038/nn.2112 18408715

[11] Logan GD, Cowan WB, Davis KA (1984) On the ability to inhibit simple and choice reaction time responses: a model and a method. Journal of Experimental Psychology. 10, 276–291. 10.1037//0096-1523.10.2.276 6232345

[12] Velmans M (2002) How could conscious experiences affect brains? Journal of Consciousness Studies. 9, 3–29.

[13] Shadlen MN, Newsome WT (1996) Motion perception: seeing and deciding. Proc Natl Acad Sci U S A. 93, 628–633. 10.1073/pnas.93.2.628 PMC40102 8570606

[14] Sebanz N Bekkering H, Knoblich G (2006) Joint action: bodies and minds moving together. Trends in Cognitive Sciences. 10, 70–76. 10.1016/j.tics.2005.12.009 16406326

[15] Otten LJ, Quayle AH, Akram S, Ditewig TA, Rugg MD (2006) Brain activity before an event predicts later recollection. Nature neuroscience. 9, 489–491. 10.1038/nn1663 16501566

[16] Busch NA, Dubois J, VanRullen R (2009) The Phase of Ongoing EEG Oscillations Predicts Visual Perception. J. Neurosci. 29, 7869–7876. 10.1523/JNEUROSCI.0113-09.2009 PMC6665641 19535598

[17] Luck SJ (2005) An introduction to the event-related potential technique, MIT Press.

[18] Fox MD, Snyder AZ, Vincent JL, Raichle ME (2007) Intrinsic Fluctuations within Cortical Systems Account for Intertrial Variability in Human Behavior. Neuron. 56, 171–184. 10.1016/j.neuron.2007.08.023 17920023

[19] Brass M, Haggard P (2007) To do or not to do: the neural signature of self-control. Journal of Neuroscience. 27, 9141. 10.1523/JNEUROSCI.0924-07.2007 PMC6672190 17715350

[20] Bradshaw JL (2001) Asymmetries in preparation for action. Trends in Cognitive Sciences. 5, 184–185. 10.1016/s1364-6613(00)01656-9 11323250

[21] Dirnberger G, Duregger C, Lindinger G, Lang W (2011) On the regularity of preparatory activity preceding movements with the dominant and non-dominant hand: a readiness potential study. Int J Psychophysiol. 81, 127–131. 10.1016/j.ijpsycho.2011.04.008 21586305

[22] Gruber MJ, Otten LJ (2010) Voluntary control over prestimulus activity related to encoding. The Journal of Neuroscience. 30, 9793. 10.1523/JNEUROSCI.0915-10.2010 PMC2929460 20660262

[23] Stroop JR (1935) Studies of interference in verbal reactions. Journal of Experimental Psychology. 18, 3–662.

[24] Wald A, Wolfowitz J (1940) On a test whether two samples are from the same population. The Annals of Mathematical Statistics. 11, 147–162.

[25] Tecce JJ (1972) Contingent negative variation (CNV) and psychological processes in man. Psychological Bulletin. 77, 73–108. 10.1037/h0032177 4621420

[26] Walter WG, Coope R, Aldrige VJ, McCallum WC, Winter AL (1964) Contingent negative variation: an electric sign of sensorimotor association and expectancy in the human brain. Nature. 203, 380–384. 10.1038/203380a0 14197376

[27] Hillyard SA (1969) Relationships between the contingent negative variation (CNV) and reaction time. Physiology & Behavior. 4, 351–357.

[28] S A S Institute (1999) SAS/STAT User’s guide: Version 8, 1SAS institute.

[29] Cheyne J, Solman GJF, Carriere JSA, Smilek D (2009) Anatomy of an error: A bidirectional state model of task engagement/disengagement and attention-related errors. Cognition. 111, 98–113. 10.1016/j.cognition.2008.12.009 19215913

[30] Robertson IH, Manly T, Andrade J, Baddeley BT, Yiend J (1997) ‘Oops!’: Performance correlates of everyday attentional failures in traumatic brain injured and normal subjects. Neuropsychologia. 35, 747–758. 10.1016/s0028-3932(97)00015-8 9204482

[31] Busch NA, VanRullen R (2010) Spontaneous EEG oscillations reveal periodic sampling of visual attention. Proc Natl Acad Sci U S A. 107, 16048–16053. 10.1073/pnas.1004801107 PMC2941320 20805482

[32] Britz J, Michel CM (2010) Errors can be related to pre-stimulus differences in ERP topography and their concomitant sources. NeuroImage. 49, 2774–2782. 10.1016/j.neuroimage.2009.10.033 19850140

[33] Mazaheri A, DiQuattro NE, Bengson J, Geng JJ (2011) Pre-Stimulus Activity Predicts the Winner of Top-Down vs. Bottom-Up Attentional Selection. PLoS ONE. 6, e16243. 10.1371/journal.pone.0016243 PMC3046127 21386896

[34] Nagai Y, Critchley HD, Featherstone E, Fenwick PBC, Trimble MR, et al.. (2004) Brain activity relating to the contingent negative variation: an fMRI investigation. Neuroimage. 21, 1232–1241. 10.1016/j.neuroimage.2003.10.036 15050551

[35] Wittmann M, von Steinbüchel N, Szelag E (2001) Hemispheric specialisation for self-paced motor sequences. Brain Res Cogn Brain Res. 10, 341–344. 10.1016/s0926-6410(00)00052-5 11167058

[36] Schlaghecken F, Eimer M (2004) Masked prime stimuli can bias“ free” choices between response alternatives. Psychonomic Bulletin and Review. 11, 463–468. 10.3758/bf03196596 15376796

[37] Hughes G, Velmans M, de Fockert J (2009) Unconscious priming of a no-go response. Psychophysiology. 46, 1258–69. 10.1111/j.1469-8986.2009.00873.x 19686367

[38] Passingham RE, Bengtsson SL, Lau H (2009) Medial frontal cortex: from self-generated action to reflection on one’s own performance. Trends in cognitive sciences. 10.1016/j.tics.2009.11.001 PMC2806969 19969501

[39] Goldberg I, Ullman S, Malach R (2008) Neuronal correlates of “free will” are associated with regional specialization in the human intrinsic/default network. Consciousness and Cognition. 17, 587–601. 10.1016/j.concog.2007.10.003 18082425

[40] Walsh E, Kühn S, Brass M, Wenke D, Haggard P (2010) EEG activations during intentional inhibition of voluntary action: an electrophysiological correlate of self-control? Neuropsychologia. 48, 619–626. 10.1016/j.neuropsychologia.2009.10.026 19883667

[41] Banks WP, Isham EA (2009) We Infer Rather Than Perceive the Moment We Decided to Act. Psychological Science. 20, 17–21. 10.1111/j.1467-9280.2008.02254.x 19152537

[42] Haggard P (2008) Human volition: towards a neuroscience of will. Nature Reviews Neuroscience. 9, 934–946. 10.1038/nrn2497 19020512

[43] Nichols S (2011) Experimental Philosophy and the Problem of Free Will. Science. 331, 1401. 10.1126/science.1192931 21415346

[44] Vohs KD, Schooler JW (2008) The value of believing in free will. Psychological Science. 19, 49. 10.1111/j.1467-9280.2008.02045.x 18181791

[45] Rigoni D, Kühn S, Sartori G, Brass M (2011) Inducing Disbelief in Free Will Alters Brain Correlates of Preconscious Motor Preparation: The Brain Minds Whether We Believe in Free Will or Not. Psychol Sci. 10.1177/0956797611405680 21515737

[46] Libet B (1985) Unconscious Cerebral Initiative and the Role of Conscious Will in Voluntary Action. Behavioral and Brain Sciences. 8, 529–539.

