# Supplementary material for: There Is No Free Won’t: Antecedent Brain Activity Predicts Decisions to Inhibit
Source: PLoS One. 2013 Feb 13;8(2):e53053. doi: 10.1371/journal.pone.0053053 (PMC3572111; doi:10.1371/journal.pone.0053053)
Supplement: Supporting Information S1 — This file contains supporting figures, S1 and S2, and table S1. (DOCX) [file pone.0053053.s001.docx]

**Supporting Information S1**

**Supplementary results**

**Figure S1**


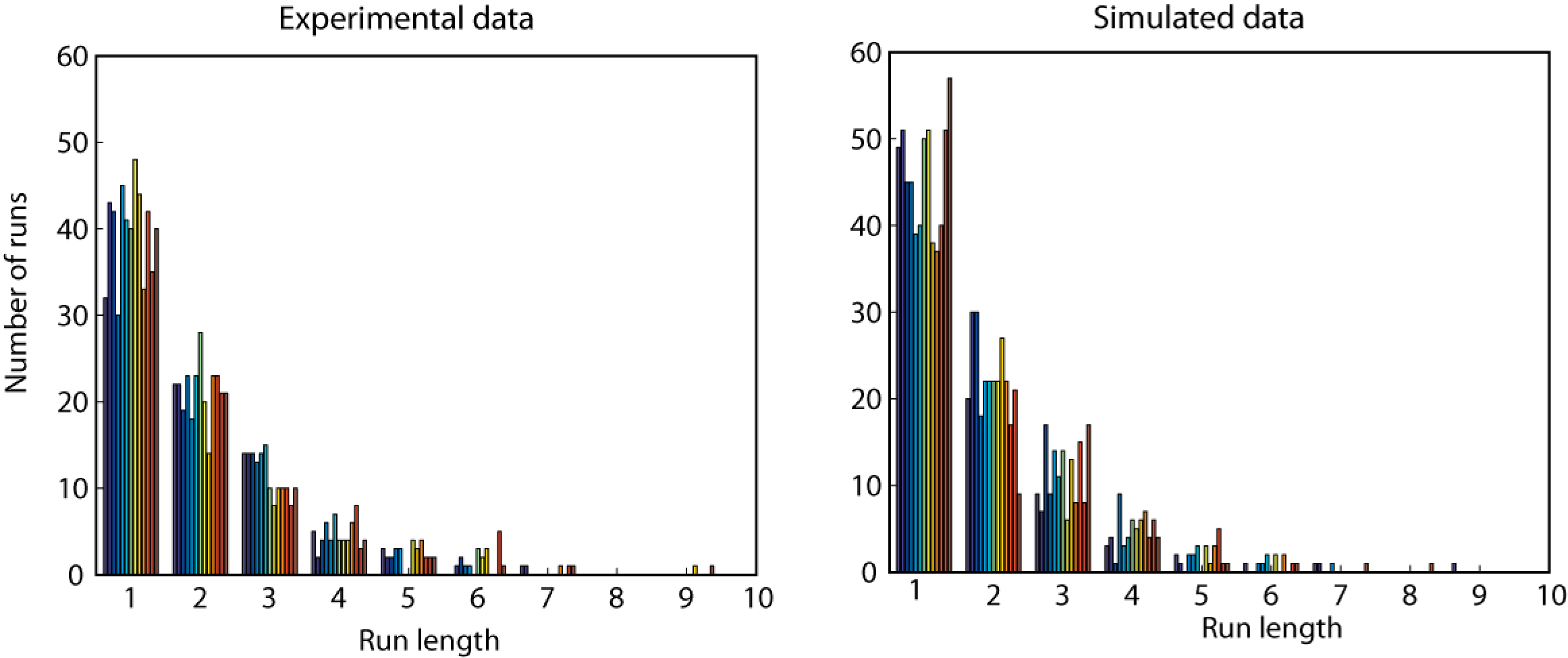


**Figure S1: Distribution of run lengths in the free-choice trials for (A) all participants and (B) simulated random data.** A run is an uninterrupted sequence of repeated choices to either press quickly or delay. Each colour represents an individual participant. Our participants tended to produce less runs of length 1 (less direct alternations) than would be expected from simulated random sequences (see main text for details)

**Figure S2**

Figure S2 shows the 95% confidence intervals for the mean ERP amplitude per condition in each time window of analysis. Results are drawn from the subsampling analysis that controls for uneven numbers of trials, as in figure 5 in the main text.


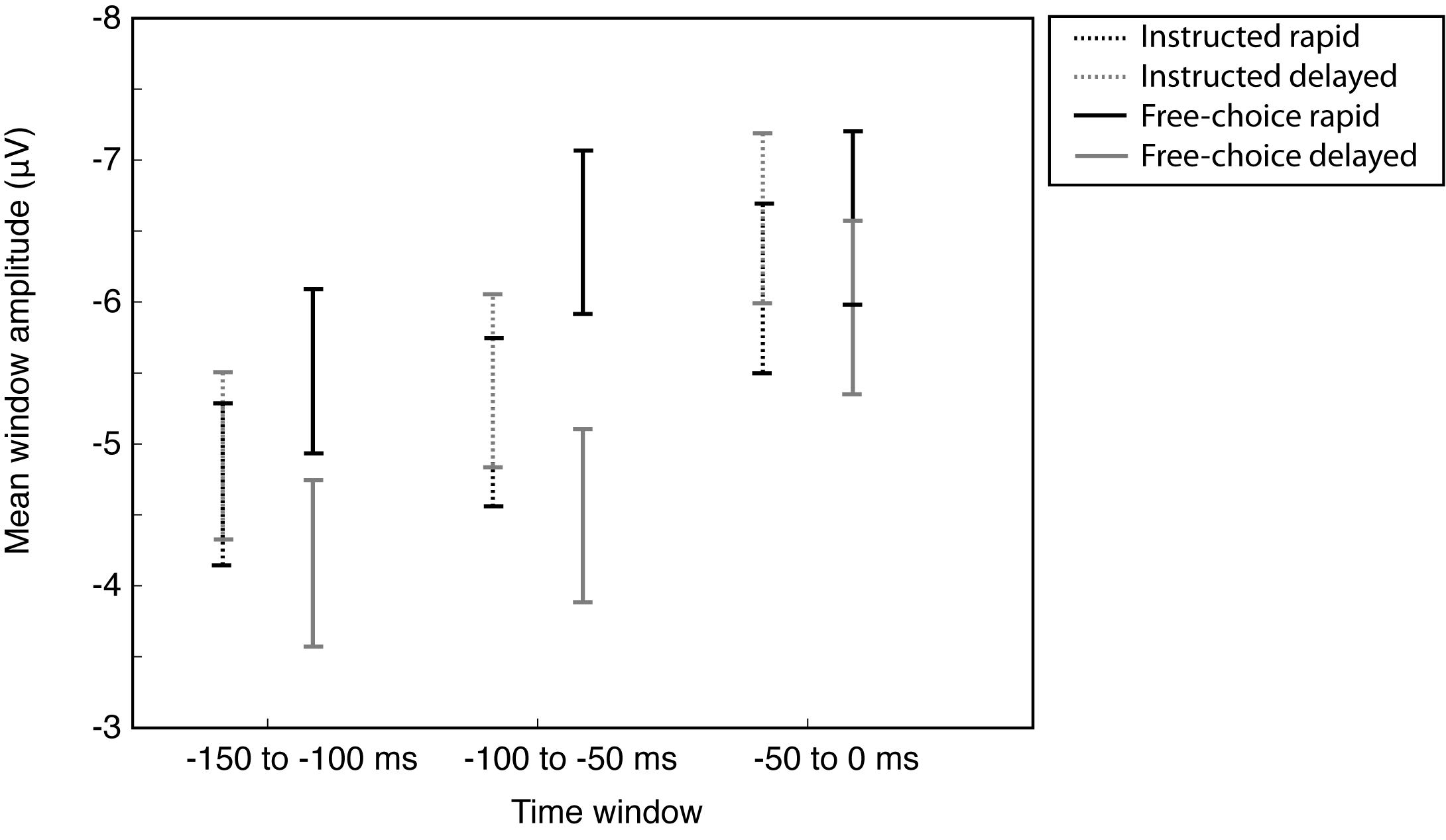


**Figure S16:** **Results of the bootstrapping procedure in each 50 ms time window analyzed.** Bars show the 95% confidence intervals of the mean ERP amplitude in Cz (as in panel B of figure 5). The first two time windows (-150 to -100 ms and -100 to -50 ms) show a significant source (instructed/free-choice) x outcome (rapid/delayed) interaction effect. Note that while the 95% confidence intervals do not overlap in the free-choice conditions, they do overlap in the instructed conditions. See table 1 for the corresponding results of statistical analysis.

**Table S1:** Mean (± standard deviation) EEG amplitudes in the three prestimulus intervals considered.

|  | **Amplitude (μV) ± SD** | | | |
| --- | --- | --- | --- | --- |
|  | **Instructed** | | **Free-choice** | |
| **Time interval (ms)** | **Rapid** | **Delayed** | **Rapid** | **Delayed** |
| **-150 to -100** | -5.03 ± 3.35 | 5.28 ± 3.42 | 5.87 ± 3.93 | -4.47 ± 4.30 |
| **-100 to -50** | -5.53 ± 4.33 | -5.82 ± 4.11 | -7.01 ± 4.14 | -4.80 ± 4.58 |
| **-50 to 0** | -6.54 ± 5.16 | -7.07 ± 5.03 | -7.04 ± 5.47 | -6.40 ± 4.89 |
