# Supplementary material for: There Is No Free Won’t: Antecedent Brain Activity Predicts Decisions to Inhibit
Source: PLoS One. 2013 Feb 13;8(2):e53053. doi: 10.1371/journal.pone.0053053 (PMC3572111; doi:10.1371/journal.pone.0053053)
Supplement: Supporting Information S2 — This file contains supporting figures, S3 to S16, representing individual distributions of RTs. (DOCX) [file pone.0053053.s002.docx]

**Supporting Information S2**

**Supplementary figures**

Figures S3 to S16 shows the individual RT distributions. For each participant, top histograms show RTs in the instructed conditions. Bottom histograms show RTs in the free choice conditions.

To encourage preparation, the experimental design included a high proportion of instructed quick trials (see Methods in the main text). The RT distributions reflect this imbalance. A bimodal RT distribution is nevertheless evident in most participants for the instructed conditions.

Free choice conditions show more uniform distributions, with smaller values of bimodality, see main text. Importantly, this argues against the possibility that the observed ERP effects are simply due to differences in RTs. In particular, a more uniform distribution in the free choice conditions would have lead to smaller (and not larger, as we observe) ERP differences between quick and delayed conditions.

**Figure S3**


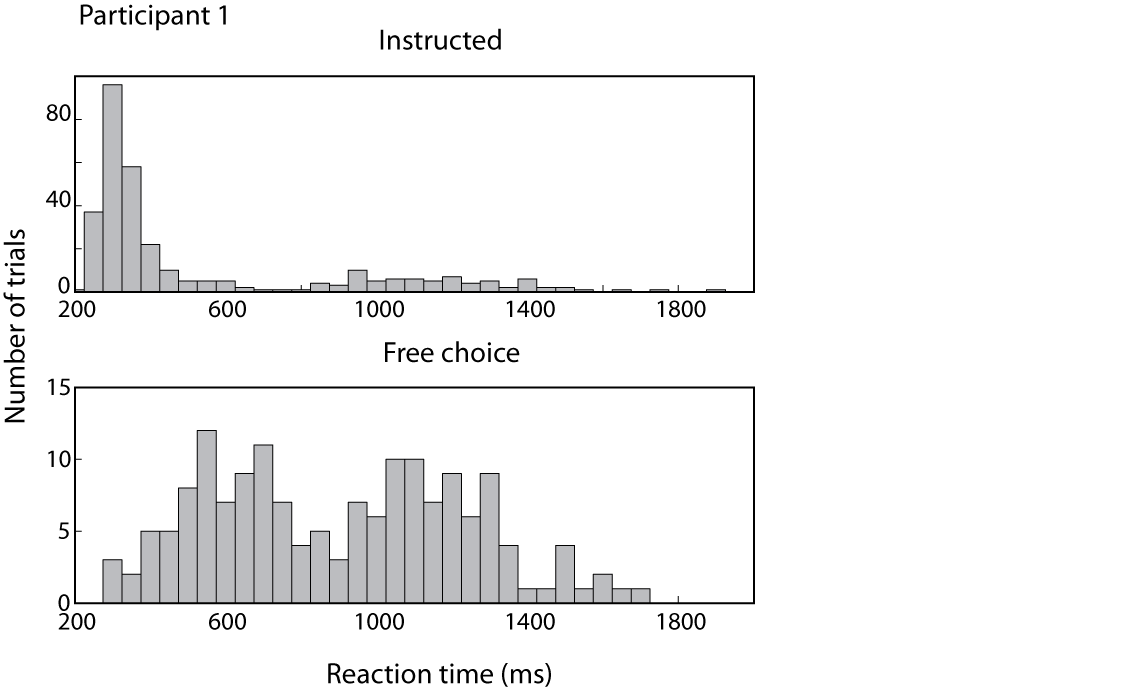


**Figure S4**


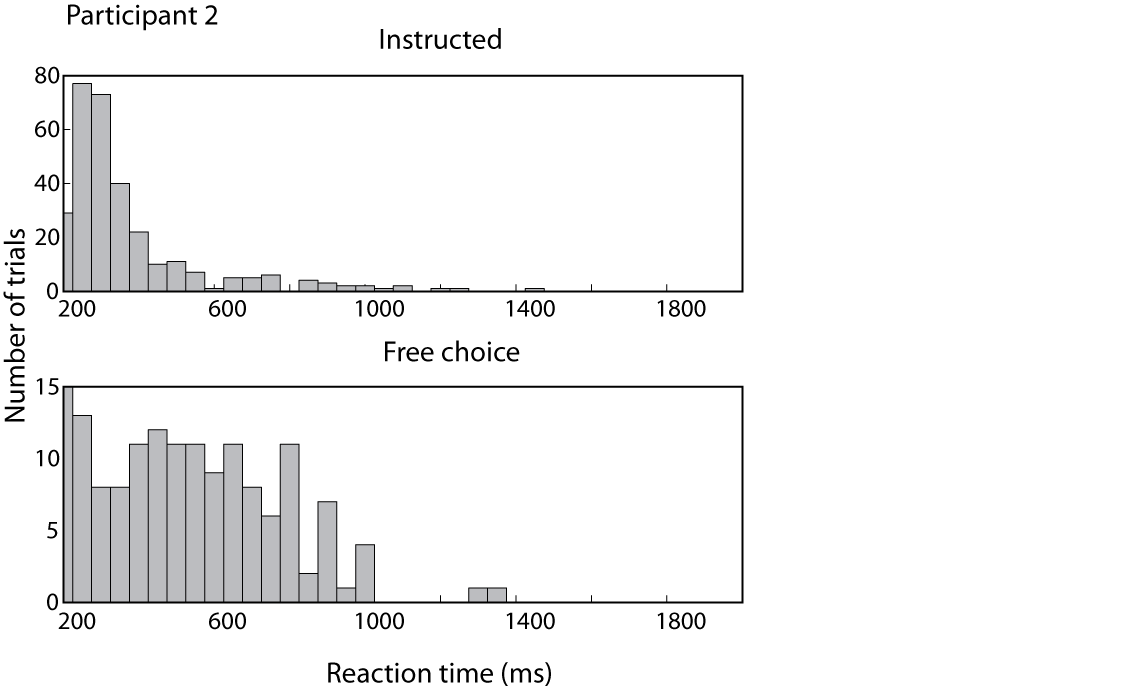


**Figure S5**


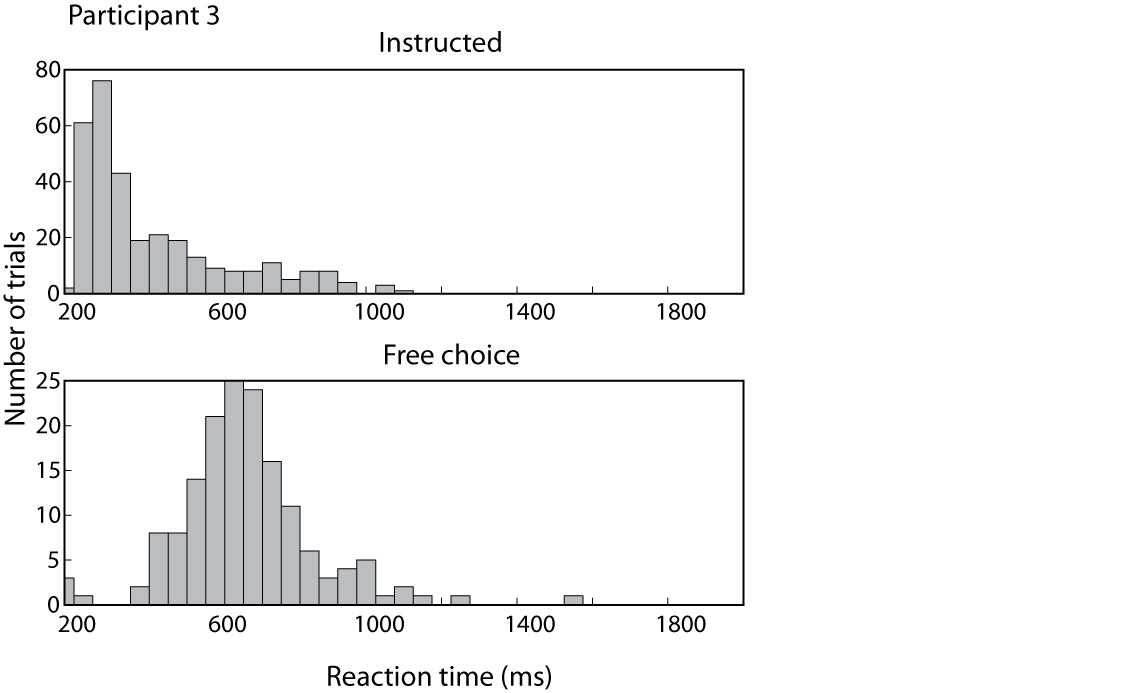


**Figure S6**


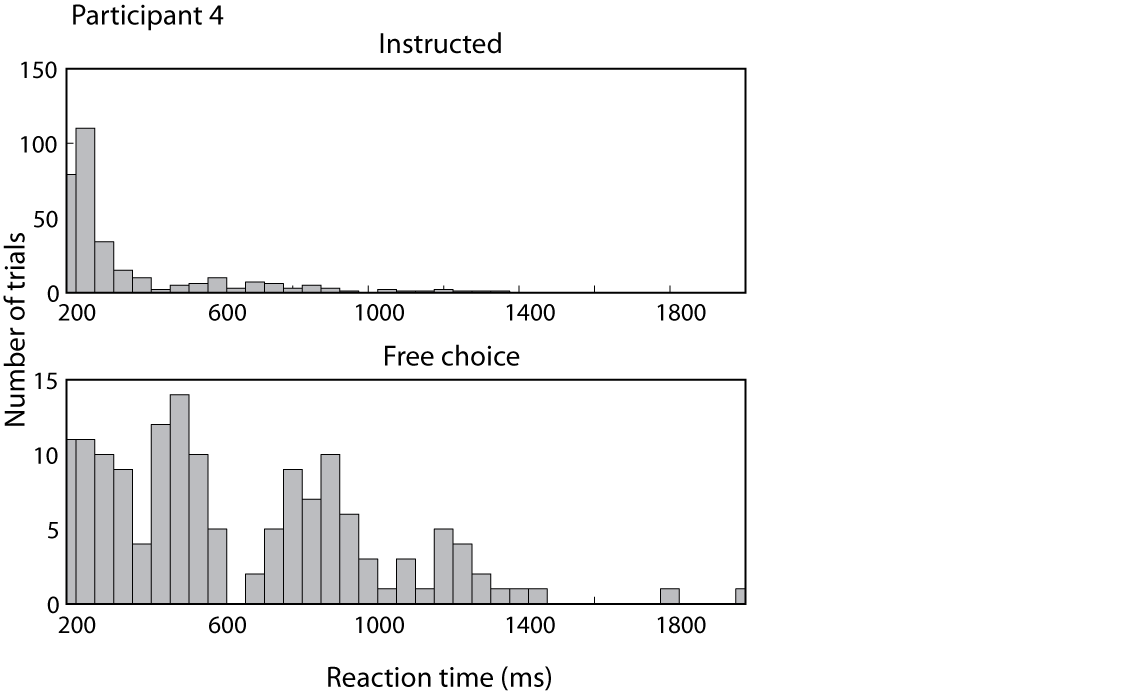


**Figure S7**


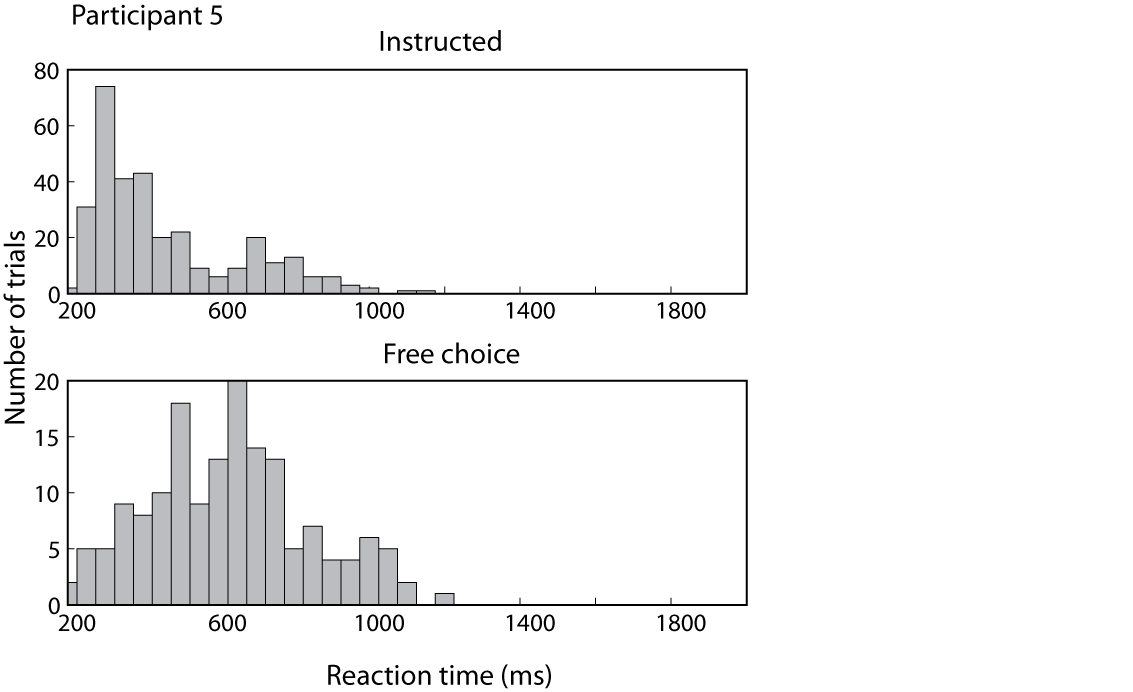


**Figure S8**


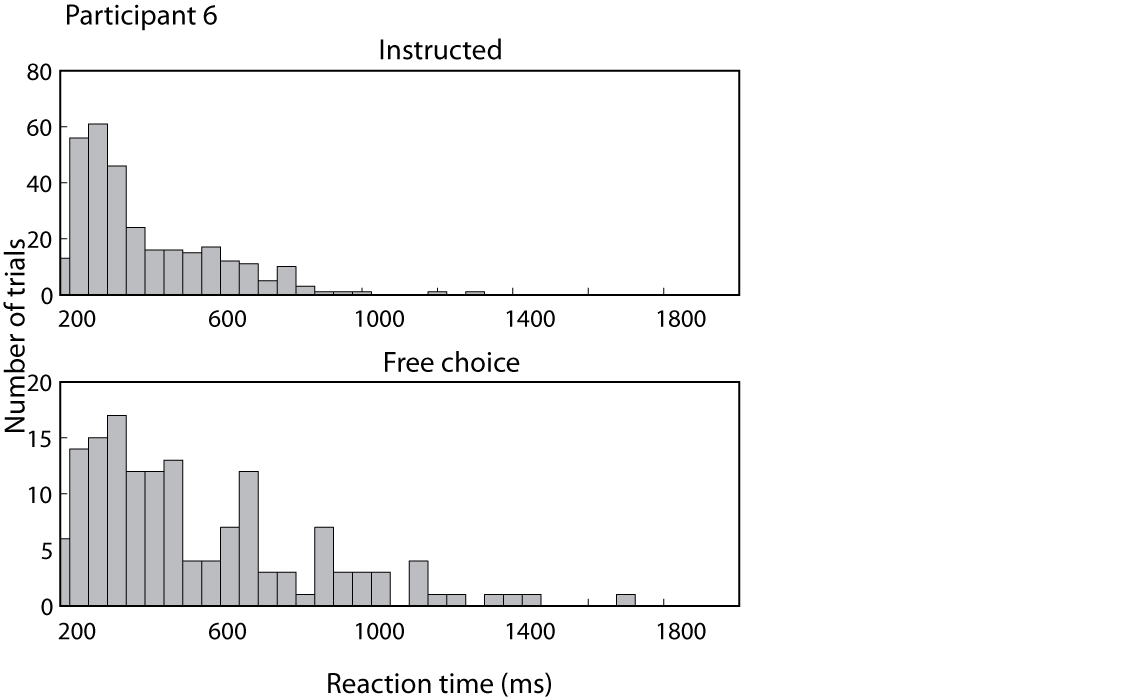


**Figure S9**


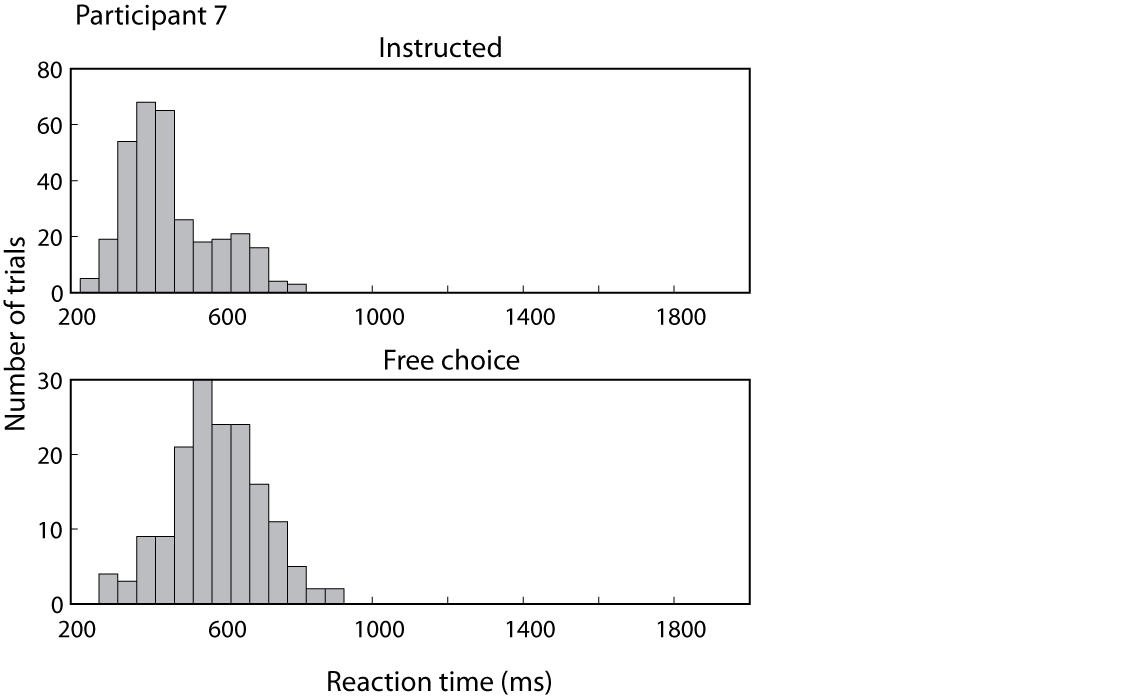


**Figure S10**


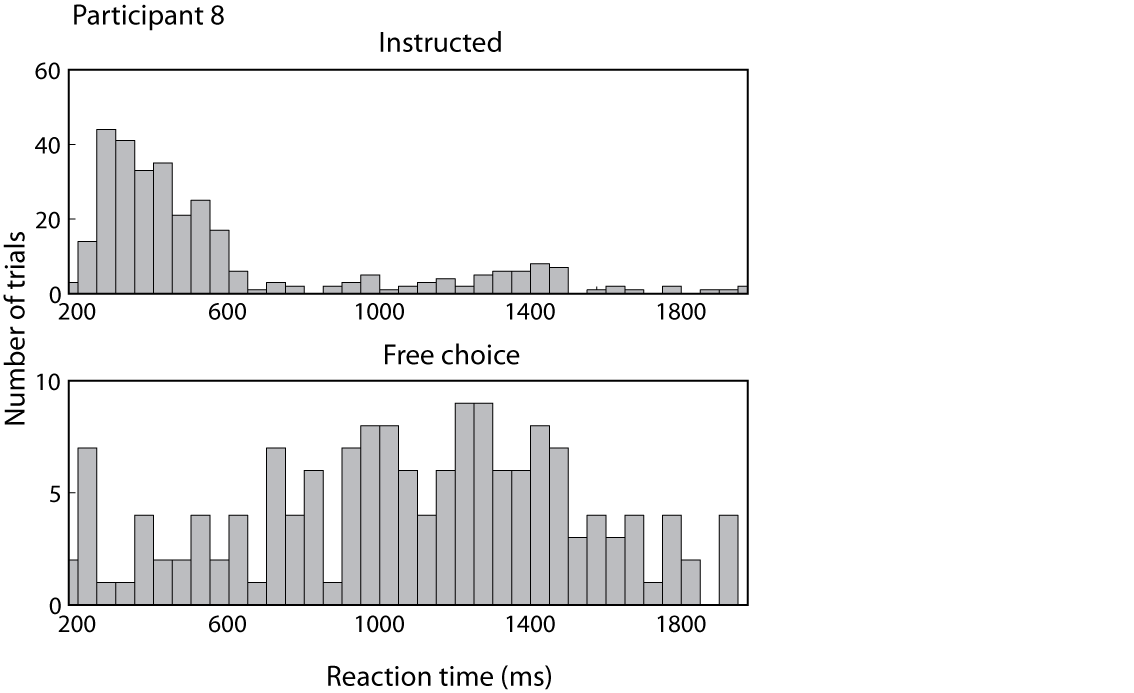


**Figure S11**


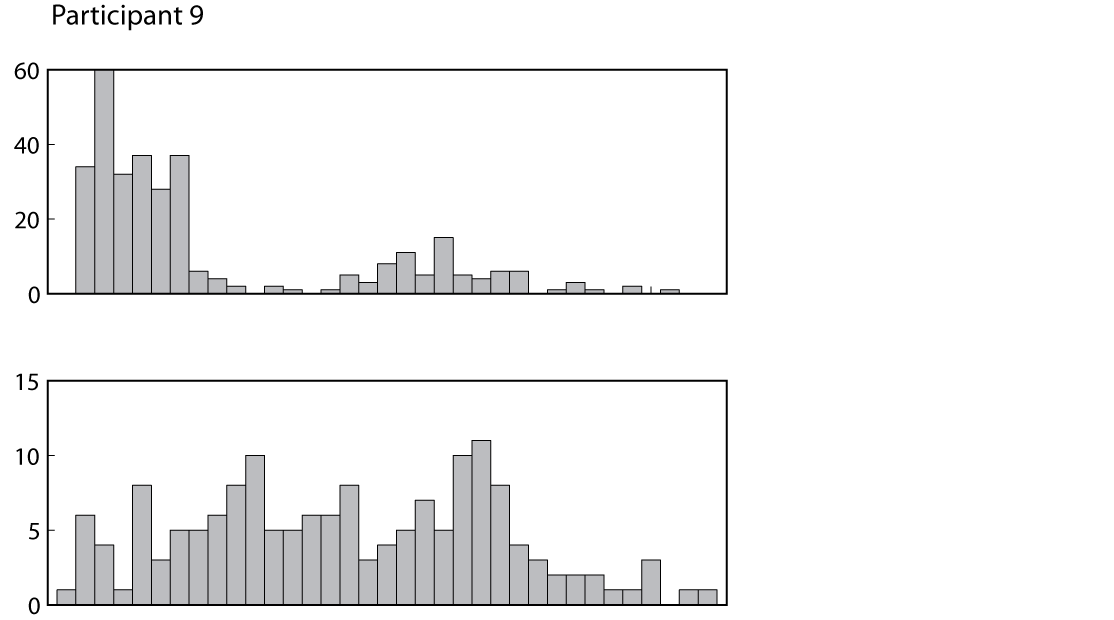


**Figure S12**


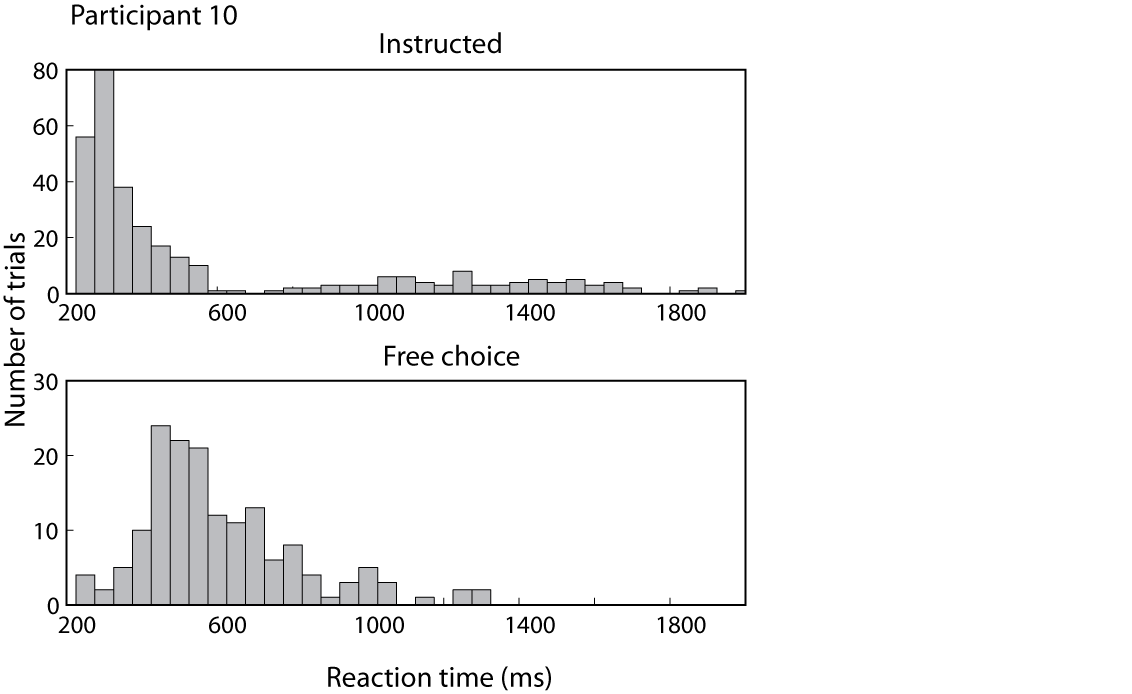


**Figure S13**


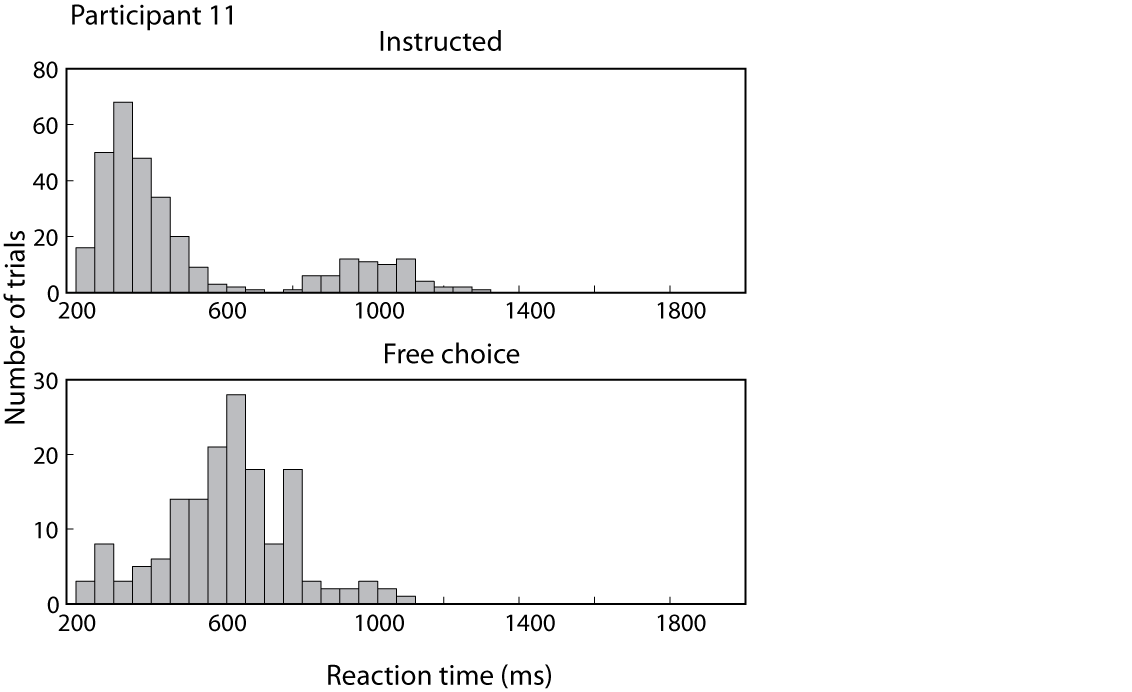


**Figure S14**


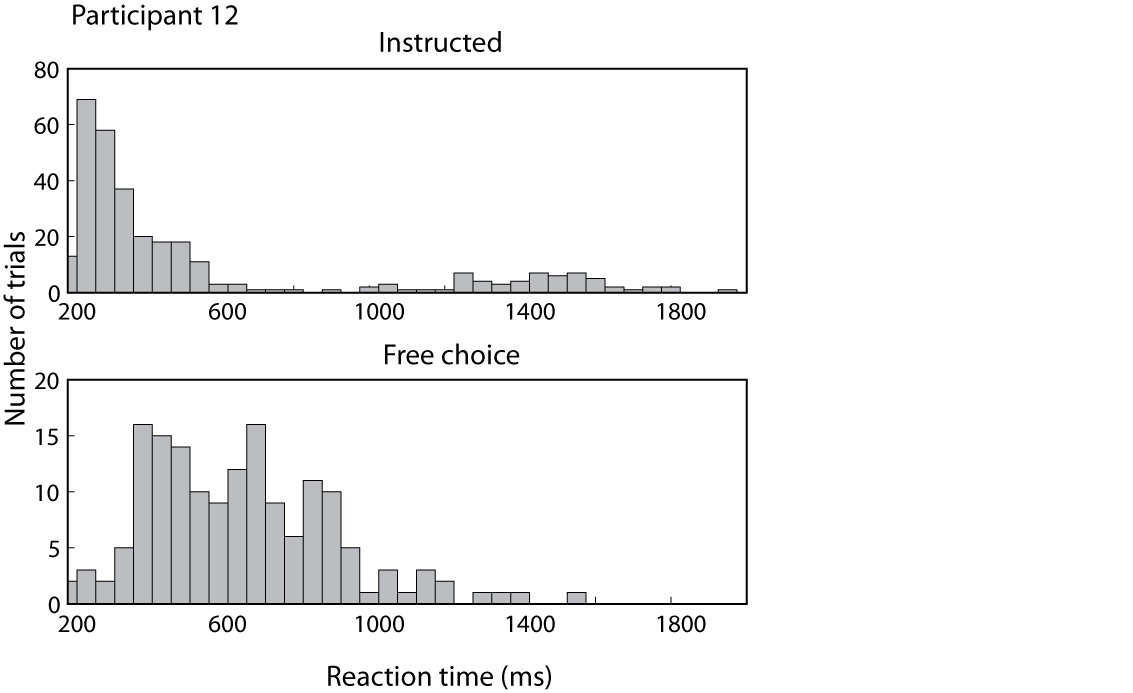


**Figure S15**


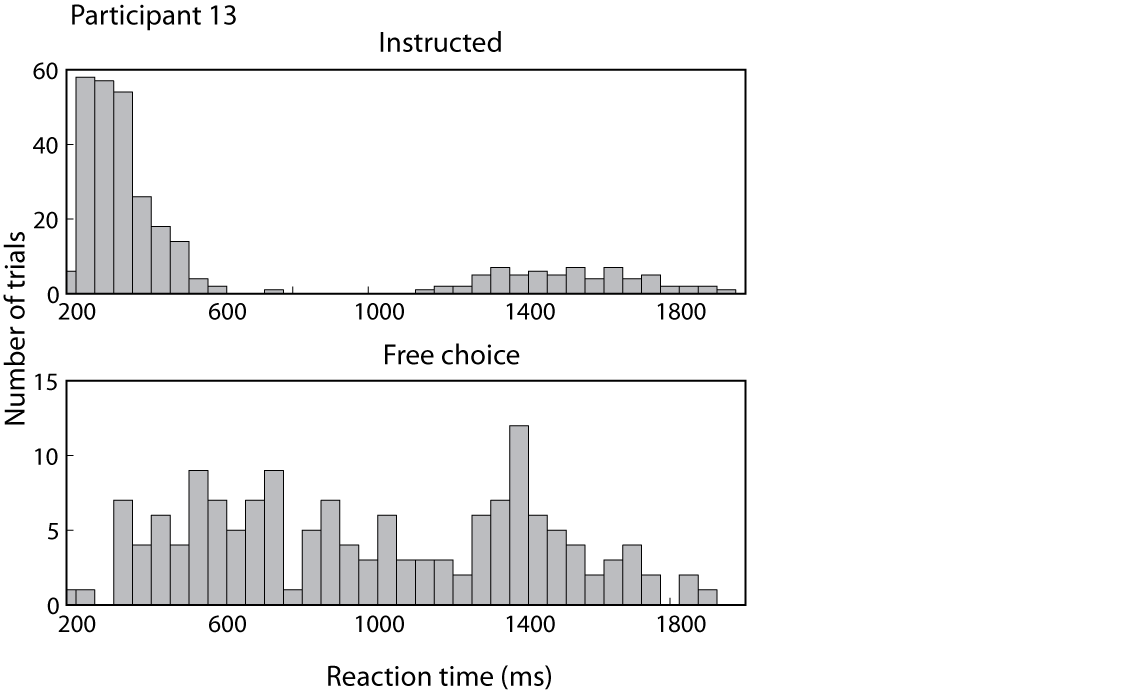


**Figure S16**


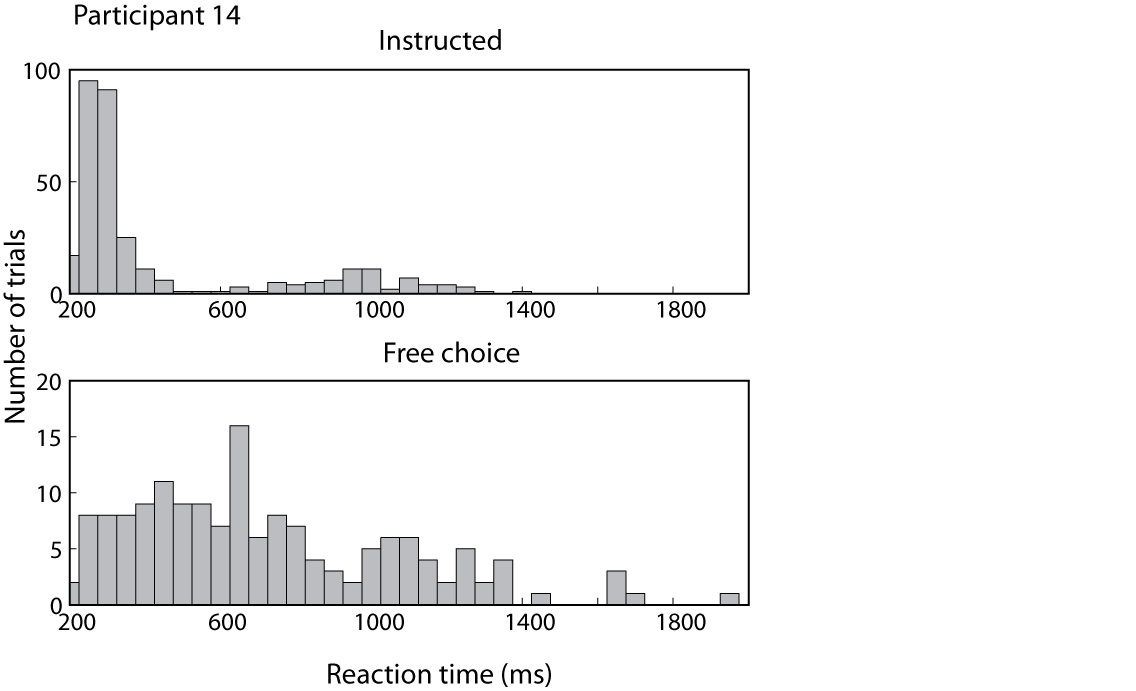


**Figures S3 to S16: Individual RT distributions for each participant.** Time bins represent 50 ms. Note that y axis values are different for each participant.
